# Nasal administration of the neuroprotective candidate NeuroEPO to healthy volunteers: a randomized, parallel, open-label safety study

**DOI:** 10.1186/s12883-017-0908-0

**Published:** 2017-07-04

**Authors:** Orestes Santos-Morales, Alina Díaz-Machado, Daise Jiménez-Rodríguez, Yaisel Pomares-Iturralde, Tatiana Festary-Casanovas, Carlos A. González-Delgado, Sonia Pérez-Rodríguez, Eulalia Alfonso-Muñoz, Carmen Viada-González, Patricia Piedra-Sierra, Idrian García-García, Daniel Amaro-González, for the NeuroEPO Study Group, Julio César García-Rodríguez, Iliana Sosa-Testé, Alicia Lagarto-Parra, Laura Barrero-Viera, Marlene David-Baldo, Maura Tamayo-Rodríguez, Ivonne Rivero-Vázquez, Gricel González-Gamiz, Alis Martín-Trujillo, Yasmila Rodríguez-Fernández, Ana Alfa Ledo-de la Luz, Maylén Álvarez-Delgado, Ivón Howland-Álvarez, Yolanda Cruz-Gómez

**Affiliations:** 1 NeuroEPO Research and Development Group, Center of Molecular Immunology, Havana, Cuba; 2 National Center for Toxicology, “Carlos J. Finlay” University Hospital, Havana, Cuba; 3 Clinical Trials Group, Research Direction, Center for Drug Research and Development (CIDEM), Ave. 26 and Puentes Grandes, No. 1605, Nuevo Vedado, Havana, Cuba

**Keywords:** Non-hematopoietic recombinant erythropoietin, NeuroEPO, Stroke, Neurodegenerative diseases, Healthy volunteers, Safety, Hematopoietic activity

## Abstract

**Background:**

Delivery of therapeutic agents as erythropoietin (EPO) into Central Nervous System through intranasal route could benefit patients with neurological disorders. A new nasal formulation containing a non-hematopoietic recombinant EPO (NeuroEPO) has shown neuroprotective actions in preclinical models. In the current study, the safety of NeuroEPO was evaluated for the first time in humans.

**Methods:**

A phase I, randomized, parallel, open-label study was carried out in healthy volunteers. They received, intranasally, 1 mg of NeuroEPO every 8 h during 4 days (Group A) or 0.5 mg of NeuroEPO (Group B) with the same schedule. The working hypothesis was that intranasal NeuroEPO produce <10% of severe adverse reactions in the evaluated groups. Therefore, a rigorous assessment of possible adverse events was carried out, which included tolerance of the nasal mucosa and the effect on hematopoietic activity. Clinical safety evaluation was daily during treatment and laboratory tests were done before and on days 5 and 14 after starting treatment.

**Results:**

Twenty-five volunteers, 56% women, with a mean age of 27 yrs. were included. Twelve of them received the highest NeuroEPO dose. Twenty types of adverse events occurred, with headache (20%) and increase of hepatic enzymes (20%) as the most reported ones. Nasopharyngeal itching was the most common local event but only observed in four patients (16%), all of them from the lowest dose group. About half of the events were very probably or probably caused by the studied product. Most of the events were mild (95.5%), did not require treatment (88.6%) and were completely resolved (81.8%). No severe adverse events were reported. During the study the hematopoietic variables were kept within reference values.

**Conclusions:**

NeuroEPO was a safe product, well tolerated at the nasal mucosa level and did not stimulate erythropoiesis in healthy volunteers.

**Trial registration:**

Cuban Public Registry of Clinical Trials RPCEC00000157, June 10, 2013.

## Background

The search of neuroprotective agents in stroke has been intended for more than 25 years to interfere with the molecular events taking place into nerve cells during or after exposure to ischemia. Nevertheless, none of them have met efficacy and safety criteria in controlled clinical trials [1, 2]. Neuroprotective actions of recombinant human erythropoietin (rHu-EPO) have been evaluated both in vitro and in vivo, demonstrating antiapoptotic, antioxidative, antiinflammatory, neurotrophic and angiogenic properties [3, 4]. However, rHu-EPO’s action on erythropoiesis could be inconvenient by triggering an increase in cardiovascular and thromboembolic events [5].

The use of EPO, similar to that produced in the brain during hypoxia, without erythropoietic but with neuroprotective activity, could be preferable [6]. Such molecule might be administered by delivery to the upper third of nasal cavity to contact both the olfactory [7] and trigeminal [8] neural pathways. This method has been reported to effectively bypass the blood-brain barrier (BBB) providing a direct connection of therapeutic proteins including EPO with the Central Nervous System (CNS) to treat neurodegenerative disorders such as Alzheimer’s and stroke while reducing systemic exposure [9, 10]. By these pathways, intranasal EPO is able to rapidly reaches many brain regions [11, 12] to effectively protect against focal cerebral ischemia [13].

Center for Drug Research and Development (CIDEM, in Spanish) developed a nasal formulation containing EPO with non-hematopoietic activity produced by the Center of Molecular Immunology (CIM, in Spanish). This formulation named NeuroEPO incorporates bioadhesive polymers and other ingredients which increase the residence time in the nasal cavity to enhance its therapeutic effect [14–16].

Mongolian gerbils treated with 30 μg of rHu-EPO by intranasal route daily during 4 days showed a lower expression of clinical signs of ischemia and edema and a better functional integrity compared with vehicle-treated animals. The molecule was detected either in the olfactory bulbs or in the cerebellum 5 min after administration [17]. Mortality of NeuroEPO-treated gerbils decreased after surgery, and the sensory and motor function was significantly improved. Histopathological mapping showed that NeuroEPO significantly reduced the delayed neuronal death in the brain [18]. NeuroEPO had a better neuroprotective effect than systemic rHu-EPO, evidenced by the significant improvement of neurological, cognitive, and histological status [19, 20]. Additionally, NeuroEPO also improved significantly neurologic behavior in rats that which underwent transitory focal ischemia, decreasing infarction area [21].

This product did not stimulate the erythropoiesis when it was administered through intranasal route in several rodent models [22, 23]. In the *Macaca fascicularis* model a 0.15% of NeuroEPO dose was determined in the cerebrospinal fluid after 15 min of intranasal administration. In this specie treatment related-changes in blood parameters were neither observed [24].

These results suggested that nasal route may be a successful, non-invasive and a safe mode to brain access for non-hematopoietic EPO, which can be used as neuroprotective agent in patients with neurological diseases. Nevertheless, is necessary to obtain primary evidences of the tolerability of NeuroEPO in humans, which will allow subsequent clinical trials to evaluate its efficacy. The present clinical investigation aims to evaluate the safety of NeuroEPO in healthy volunteers using two dosing schedules by nasal route.

## Methods

A phase I, randomized, uncontrolled, parallel, open-label safety study was carried out at the National Center for Toxicology, in Havana, a certified reference unit for this type of studies.

### Subjects

Cuban citizens of both genders, aged between 18 and 40 years, without organic or psychological diseases in the questioning and non-symptoms or signs at physical examination and laboratory tests were included in the trial. The absence of HIV and hepatitis B and C virus infection markers in serum was required. Exclusion criteria were: women who are pregnant or breastfeeding, hypersensitivity to EPO or to any other of the ingredients of the formulation, rhinitis, nasal septum deviation, mental disorders, history of alcoholism, record of chronic diseases, treatment with any drug in the previous 15 days, surgical intervention in the previous 6 months, blood donations in the previous 3 months and participation in a clinical trial during the prior 6 months. No more than 14 days between pre-screening and the beginning of the trial were allowed. Subjects could withdraw from the trial voluntarily, due to occurrence of severe adverse events, or by the appearance of any exclusion criteria.

### Hypothesis and treatment

In this study was expected that after intranasal administration of NeuroEPO the frequency of severe adverse events certainly caused by the product was less than 10%.

NeuroEPO [CIMAB S.A, Havana, Cuba] was a stabilized liquid formulation, multidose vials, containing 6 mg (1 mg/mL) of non-hematopoietic rHu-EPO, produced in Chinese hamster ovary (CHO) cells. Each vial also contains buffer salts, polysorbate 80, sodium EDTA, NaCl, benzalkonium chloride, HPMC F4 M, and water for injection to complete 6 mL. A placebo formulation containing the same ingredients (except EPO) was also used.

Before administration, vials were kept at rest room temperature during 50 min. After this time, vials were gently shaken in form of eight, to guarantee homogenization before volume extraction. A graduated type-insulin syringe was used to administer the prefixed doses.

The Maximum Safe Starting Dose (MSSD) in healthy humans was calculated according to established guidelines [25]. This dose, estimated from the whole preclinical data, was 3.3 mg for 60 kg average bodyweight. Due to practical reasons MSSD was 3 mg daily for this clinical trial. A second dose of daily 1.5 mg was also evaluated. Taking into account that intranasal drug administration capacity is limited by the maximum volume that can be used [26, 27], it was decided to divide doses into three administrations (every 8 h). These treatments were extended for 4 days considering the data from ischemia models, acute and sub-acute toxicology and previous clinical trials with rHu-EPO [17–23, 28, 29].

Subjects were distributed according to a computer-generated simple random number list to two groups of treatment with 15 individuals each one. Subjects from Group A received 1 mg of NeuroEPO every 8 h during 4 days by nasal route. Subjects included in Group B received 0.5 mg of NeuroEPO with the same schedule, by the same route of administration. Each multidose NeuroEPO vial could be used in five subjects from Group A and ten subjects from Group B.

For each group, doses were given in two moments with the same volumes. For Group A, firstly, a volume of 250 μL (0.25 mg) of NeuroEPO into each nostril was applied and 15 min later the same application was repeated to obtain a final volume of 500 μL (0.5 mg) in each nostril. For Group B, firstly, a volume of 250 μL (0.25 mg) of NeuroEPO into each nostril was applied and 15 min later 250 μL of placebo were administered into each nostril to obtain a final volume of 250 μL (0.25 mg) in each nostril. These procedures were carried out daily at 8:00 am, 4:00 pm and 12:00 am in all the individuals.

The products were administered slowly into one of the nostrils, drop by drop, to assure a full instillation into the nasal mucous. The subjects were lying in *decubitus supine* position, with the head dorsally bowed 45 degrees from the axis of the body, to guarantee the product bypass the BBB and reach their site of action. At the same time a pressure in the opposite nostril was exerted. After this, the volunteers rested 1 min and the method was applied in the other nostril. They were requested to sustain a normal breathing during the process.

Other concomitant treatments could be administered to mitigate adverse events, after medical consent. None of these treatments could affect the results by interactions or direct effects on the tested safety variables.

Volunteers were regularly checked for vital signs and adverse manifestations during the study. They were hospitalized during the 4 days of treatment and were given discharge the following day, after evaluation. Two weeks after beginning the treatment, final evaluation was done under outpatient conditions.

### Safety evaluation

Tolerability was monitored during the whole study by means of adverse events control. Data related to adverse events were obtained through questioning or were spontaneously referred by the subject. When the event was presented, the medical investigator acted according to their nature and severity taking the required actions (pharmacological or not) for their reduction and elimination.

Events were considered severe if produce subject’s death, threatens subject’s life, requires or prolongs hospitalization or produce a significant or persistent disability. Additionally, those events that required medical or surgical intervention to prevent the occurrence of bronchial allergic spasm at home, blood dyscrasias and seizures that do not provoke hospitalization were considered as severe.

The medical terminology for adverse events and their intensity classification (grades 1–5) was applied according to the Common Terminology Criteria for Adverse Events [30]. The causal relationship was classified as very probable/certain, probable (likely), possible, unlikely, not related or unassessable/unclassifiable, according to WHO criteria for causality [31].

Blood samples were taken for hematological and biochemical determinations before (day 0) and after treatment (day 5, day 14). Hematological counts (reticulocytes, hemoglobin, hematocrit, leukocytes), coagulation parameters (platelet count, partial thromboplastin and prothrombin times) and blood chemistry (glycemia, creatinine, urea, liver enzymes) were done according to usual clinical laboratory procedures at the Clinical Laboratory of the Center for Medical-Surgical Research, Havana, Cuba, a laboratory certified by the Cuban Regulatory Agency. Advanced automated analyzers (Mindray, Shenzhen, China; Cobas, Roche Diagnostics, Basel, Switzerland) were used for these purposes. After treatment, those values outside reference limits established by this laboratory were considered as adverse events excepting transient and very close variations without clinical relevance. Laboratory evaluations were done blindly regarding the subject’ group allocation.

Before and after each administration and in each evaluation time, vital signs taking and physical examination were carried out. The presence of toxicity signs in the nasal mucous, such as: redness, swelling and nasal congestion was evaluated by means of thorough medical examination of the nasal cavity by the same Otorhinolaryngology Specialist.

### Statistical analysis

Sample size was determined in correspondence with the aim of the study, the international trend in this type of study, the predominant descriptive nature of the analysis of the variables and the need to minimize the number of subjects exposed to the investigational medicinal product [32, 33]. A sample size of 30 subjects (15 per group) was chosen. The possibility of compensating withdrawals was not foreseen.

Data were double entered and validated and then imported into SPSS for Windows (version 15.0, IBM Analytics 2006, Armonk, North Castle, NY, USA) and Epidat (version 3.1, Directorate General of Public Health (Xunta de Galicia) 2006, Santiago de Compostela, Spain) for further analysis. Continuous variables were expressed as mean ± standard deviation (SD) or median ± interquartile range (QR) and categorical variables (e.g. adverse events) were given as frequencies and percentages. For laboratory measurements and vital signs normality analysis (Kolmogorov-Smirnov’s test or Shapiro Wilk’s test) and homogeneity of variance (Levene’s test) were carried out. These variables were analyzed through paired analysis (non-parametrical Wilcoxon’s test) comparing initial values with those obtained on 5th and 14th day for each group. Additionally, groups were compared at each time using the Mann-Whitney’s U test. Significance level was 0.05.

## Results

**Fig. 1:**
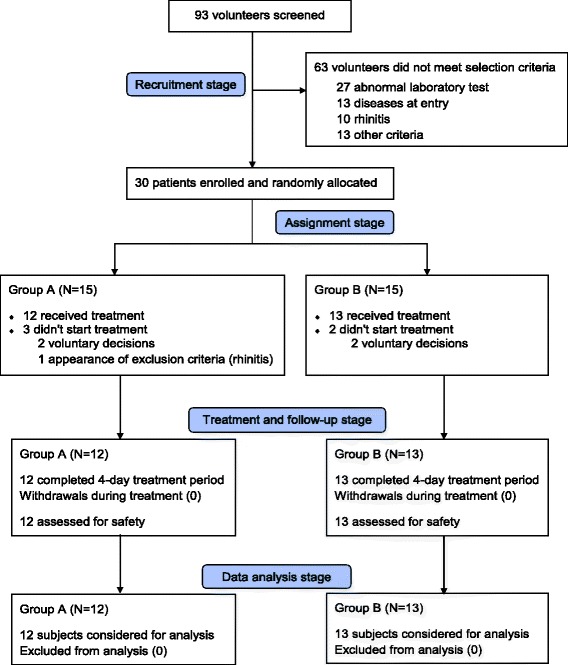
Trial summary

After medical check-up, 30 apparently healthy volunteers were selected among a universe of 93 subjects who expressed their consent to participate in the study (Fig. 1). The causes of no inclusion were: abnormal clinical laboratory values (27 subjects), presence of organic or psychic disease (13), presence of rhinitis (10), blood donation in the previous 3 months (3), nasal septum deviation (7) and voluntary abandonment before inclusion (3).

Included volunteers were randomly assigned to one of the two treatment groups (15 per group). However, five withdrawals, three of them from Group A, occurred prior to first dose, four were voluntary abandonment and the other case was the appearance of an exclusion criterion (rhinitis). Consequently, 25 subjects, 12 from Group A and 13 from Group B received NeuroEPO treatment. This final sample was big enough to study the hypothesis. During the study, there were no losses in the follow-up and evaluation and analysis included all subjects (Fig. 1).

Groups of treatment were homogenous according to demographic and baseline characteristics as shown in Table 1. Women slightly prevailed (56%) and white and non-white skin color proportions were similar. The subjects weighed around 65 Kg and were 167 cm tall; the mean age was 27 years.

**Table 1:** Demographic and baseline characteristics of the subjects who received 1 mg (Group A) or 0.5 mg (Group B) of NeuroEPO every 8 h, during 4 days, by nasal route

Characteristic	Group A *N* = 12	Group B *N* = 13	Total *N* = 25
Female gender	7 (58.3%)	7 (53.8%)	14 (56.0%)
Skin color			
White	7 (58.3%)	6 (46.2%)	13 (52.0%)
Non-white	5 (41.7%)	7 (53.8%)	12 (48.0%)
Age (years)	28 ± 6	26 ± 4	27 ± 5
Weight (Kg)	67 ± 13	63 ± 10	65 ± 11
Height (cm)	164 ± 9	169 ± 11	167 ± 10

Data are reported as number of subjects (%) or mean ± standard deviation

Eighty percent of treated subjects reported at least one clinical manifestation (local or systemic) or laboratory alteration (Table 2). Individuals with adverse events were two thirds in Group A and 92.3%. in Group B. Sixteen types of adverse events occurred during the trial, 13 recorded during clinical examinations and three identified during the monitoring of hematological and biochemical parameters. There were seven types of events in Group A and 12 in Group B. Upper respiratory tract events (related with nasal administration) prevailed in Group B as observed. The groups had in common three types of events: nasal mucous ardor, headache and increase of liver enzymes.

**Table 2:** Frequency of adverse events during the study

Adverse event	Group A *N* = 12	Group B *N* = 13	Total *N* = 25
Any adverse event	8 (66.7%)	12 (92.3%)	20 (80.0%)
Site of administration (local events)			
Nasopharyngeal itching	--	4 (30.8%)	4 (16.0%)
Nasal mucous ardor	1 (8.3%)	2 (15.4%)	3 (12.0%)
Sneezing	--	2 (15.4%)	2 (8.0%)
Reddened nasal mucous	--	1 (7.7%)	1 (4.0%)
Systemic events			
Headache	2 (16.7%)	3 (23.1%)	5 (20.0%)
Fever	2 (16.7%)	--	2 (8.0%)
Arterial hypertension	1 (8.3%)	--	1 (4.0%)
Diarrheas	--	1 (7.7%)	1 (4.0%)
Pruritus	--	1 (7.7%)	1 (4.0%)
Colics	--	1 (7.7%)	1 (4.0%)
Epicondylitis	1 (8.3%)	--	1 (4.0%)
Insomnia	--	1 (7.7%)	1 (4.0%)
Cough	--	1 (7.7%)	1 (4.0%)
Laboratory alterations			
Hepatic enzymes increased^a^	2 (16.7%)	3 (23.1%)	5 (20.0%)
Anemia^b^	--	2 (15.4%)	2 (8.0%)
Platelet count decreased^c^	1 (8.3%)	--	1 (4.0%)

Data are presented as number of individuals with each adverse reaction (%)

^a^ALT > 41 U/L (men) or >33 U/L (women); AST > 40 U/L (men) or >32 U/L (women); GGT > 60 U/L (men) or >40 U/L (women)

^b^Hgb: < 130 g/L (men) or <120 g/L (women)

^c^ < 150 × 10^9^ cells/L

The most frequent adverse events were headache and increase of liver enzymes, but both were reported in only two subjects from Group A and three subjects from Group B. Nasopharyngeal itching was the most common local event, only detected in four volunteers from Group B. Other events, such as: nasal mucous ardor, sneezing, fever and anemia occurred in two or three subjects in general. The rest of events, mostly systemic, were recorded in a single subject of one or the other group (Table 2).

The number of adverse events was also superior in the Group B since 61.4% of the 44 reports arose in this group (Table 3). One subject from Group B had the maximum number of reports with seven. Non-severe adverse events were recorded, thus no subject withdrew from the trial due to adverse reactions. Regarding intensity, events were mostly (95.5%) classified as grade 1 (mild). Only two events (headache and epicondylitis), both in patients from Group A, were classified as grade 2 (moderate).

**Table 3:** Characterization of the adverse events registered

Characteristic	Classification	Group A	Group B	Total
Number of events		17 (38.6%)	27 (61.4%)	44 (100%)
Severity	Non-severe	17 (100%)	27(100%)	44 (100%)
Intensity	Grade 1	15 (88.2%)	27 (100%)	42 (95.5%)
	Grade 2	2 (11.8%)	--	2 (4.5%)
Causality	Certain	--	11 (40.7%)	11 (25.0%)
	Probable	8 (47.1%)	2 (7.4%)	10 (22.7%)
	Possible	7 (41.1%)	5 (18.5%)	12 (27.3%)
	Unlikely	--	4 (14.8%)	4 (9.1%)
	Not related	2 (11.8%)	4 (14.8%)	6 (13.6%)
	Unclassifiable	--	1 (3.7%)	1 (2.3%)
Conduct	Pharmacotherapy	3 (17.7%)	1 (3.7%)	4 (9.1%)
	Other intervention	--	1 (3.7%)	1 (2.3%)
	Observational	14 (82.3%)	25 (92.6%)	39 (88.6%)
Result	Resolved	14 (82.3%)	22 (81.5%)	36 (81.8%)
	Improved	1 (5.9%)	--	1 (2.3%)
	Persisted	2 (11.8%)	5 (18.5%)	7 (15.9%)

Data are reported as number of events (%)

The number of events with certain or probable causal relationship rounded 50%. In the Group A 47% of the events had a probable relation, none certain, whereas in Group B 41% of the events were certainly caused by the product. These last ones were those produced at the site of administration. Only some systemic events or laboratory alterations (mostly in Group B) were unlikely or not related to NeuroEPO treatment. Just one increase of hepatic enzymes was unclassifiable (Table 3).

Most of the events did not require treatment (88.6%) and were well solved (81.8%). It was necessary to administer dipyrone to treat headache and fever. Ibuprofen was used to treat epicondylitis. Only seven events, five of them in the Group B, persisted at the end of the study, but these were mostly alterations in laboratory parameters which returned spontaneously to normal values, few weeks later.

Vital signs did not change significantly during the study in both groups (data non-shown). Otorhinolaryngological study proved normality in most of the subjects throughout the study, except for some individuals with the above-mentioned local events. Physical examination was also normal after the 4 days of treatment (day 5) as well as on the final evaluation (day 14).

**Fig. 2:**
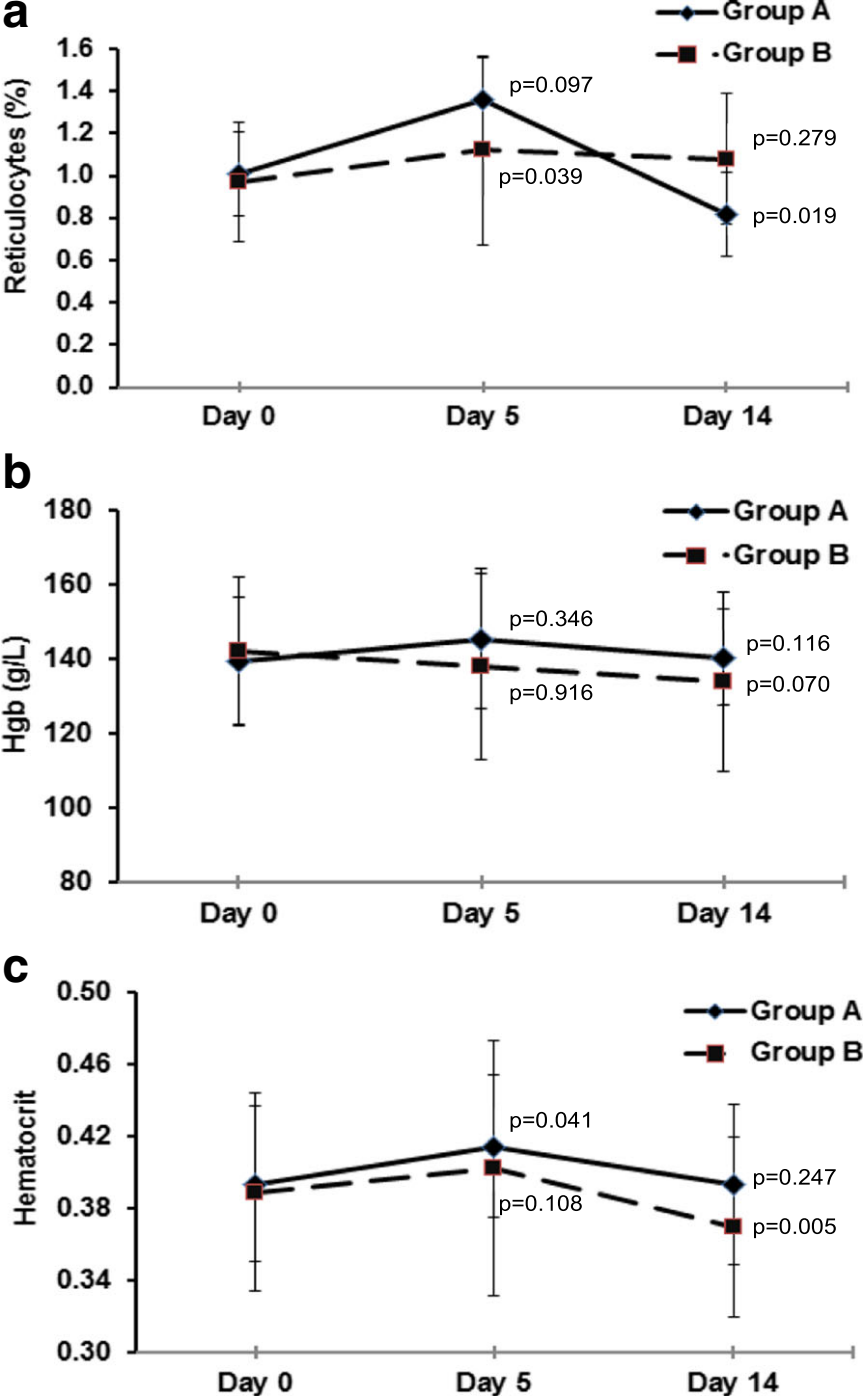
Hematological parameters before and after treatment with NeuroEPO. Data correspond to the healthy subjects who received 1 mg of NeuroEPO (Group A, *N* = 12) or 0.5 mg of NeuroEPO (Group B, *N* = 13) every 8 h, during 4 days, by nasal route. Points correspond to median and deviations for each measure before treatment (day 0) and after it (day 5, day 14). **a** Reticulocytes count (0.5–1.5%). **b** Hemoglobin (M: 130–175 g/L; W: 120–165 g/L). **c** Hematocrit (M: 0.41–0.54; W: 0.37–0.47). Post-treatment vs. initial analysis (Wilcoxon’s test) is showed for the three variables. Differences between groups of treatment were no significant at each time (*p* > 0.05, Mann-Whitney’s U test)

Concerning hematological toxicity, values in both evaluation times were kept within normal ranges (Fig. 2, see legend). Some changes detected in reticulocytes count (Fig. 2a) and hematocrit Fig. 2c) had non-clinical significance. For hemoglobin, there were no significant changes throughout the study (Fig. 2b). A mild anemia was recorded in two subjects from Group B, whose values descended to 115 and 97 g/L, respectively, on day 14. Differences between groups of treatment were no significant at each time. Other clinical laboratory measurements were not markedly affected (data non-shown).

## Discussion

The findings support the hypothesis that the frequency of severe adverse events following intranasal administration of NeuroEPO would be less than 10% since no severe events occurred. This is in accordance with the literature since the use of rHu-EPO in healthy subjects apparently does not affect physiological indexes and its use is safe for experimental purposes [34].

The current first NeuroEPO-in-human trial demonstrated the absence of hematopoietic activity according to the safety profile. This result is expected due to this EPO possesses a low content of sialic acid, a component that plays a key role in the preservation of EPO structure thereby avoiding its destruction by the liver. Low sialic acid content rHu-EPO molecules are rapidly metabolized by the liver and therefore eliminated without being able to exert their hematopoietic action [3]. This is the main expected safety benefit of NeuroEPO and has already been demonstrated in laboratory animals since the product did not modify hematopoietic activity even when used at high doses [23].

A good local tolerance was evidenced. The mild local adverse events described correspond to those observed in the preclinical studies both with NeuroEPO and controls in the nasal irritation test [23] and could also be considered common when this route of administration is used. The recorded events support the role of trigeminal pathway in the entrance of NeuroEPO to CNS. Sensory nerves of the afferent trigeminal system including myelinated Aδ-fibres and thin, non-myelinated C-fibres of the nasal mucosa transmit signals generating sensations, including itching and motor reflexes, such as sneezing [35]. The trigeminal nerve is also the primary nerve involved in headache [36].

Although rHu-EPO increases blood pressure in patients with chronic renal failure and cancer [37, 38], the mild event observed in one subject could be considered an isolated event within the framework of the study, considering that the rest of subjects preserved normal values.

A mild rise of the liver enzymes values was founded in some individuals. There is no history of increase of these enzymes in acute toxicological studies with NeuroEPO. However, considering temporal relationship between NeuroEPO administration and the appearance of the event, this aspect should continue being studied as part of the clinical development of the product.

Considering that Group B received the lowest NeuroEPO dose, a direct relationship between dose and the frequency of adverse events was not evidenced. Later studies will confirm or not if patients treated with smaller doses have a higher frequency of local events. The contribution of formulation components to these adverse events should be also considered.

Erythropoietin has been proposed for neuroprotection [39]. It has shown to have more than one mechanism of action, and continues to be a promising choice in the future, concerning the data review of brain ischemia models [40]. This molecule is able to reach CNS minimally between 9 and 24 h after intravenous administration [41]. The first clinical evidence of the utility of rHu-EPO in the treatment of stroke was obtained in the early 2000s [28]. A further attempt to replicate this outcome failed because of safety concerns that the authors associated with errors in the execution of the trial [29].

Nasal administration provides a promissory route of administration for EPO [42]. Intranasal rHu-EPO was able to recover spontaneous motor activity, without induction of peripheral erythropoiesis in a focal brain hypoxia model [43]. The information now obtained indicates us a dose frame to which rHu-EPO can be safely administered through nasal route. The NeuroEPO dosing scheme was previously validated in a cerebral ischemia model improving neurological status and increasing viability and spontaneous exploratory activity, also showed a therapeutic window up to 12 h [20]. Moreover, this product confirmed their high neuroprotective activity since it relieved memory alterations, oxidative stress, neuroinflammation, apoptosis induction and amyloid load in a reference transgenic mouse model of Alzheimer’s disease [44].

The results in healthy volunteers justified the approval of further clinical trials with NeuroEPO formulation in stroke [45] and spinocerebellar ataxia [46]. These trials are ongoing and preliminary results in ataxia are encouraging.

## Conclusions

Intranasal administration of NeuroEPO in healthy volunteers was well tolerated without undesired hematopoietic effects. These results strongly validate the continuity of clinical development of this product in patients with stroke and neurodegenerative diseases.

## Abbreviations

ALT: Alanine aminotransferase
AST: Aspartate aminotransferase
BBB: Blood-brain barrier
CHO: Chinese hamster ovary
CNS: Central Nervous System
EDTA: Ethylenediaminetetraacetic acid
GGT: Gamma-glutamyltransferase
Hgb: Hemoglobin
HPMC: Hydroxypropyl methylcellulose
MSSD: Maximum safe starting dose
NaCL: Sodium chloride
rHu-EPO: Recombinant human erythropoietin
WHO: World Health Organization
